# Comparison between lentigo maligna melanoma and other histogenetic types of malignant melanoma of the head and neck. Scottish Melanoma Group.

**DOI:** 10.1038/bjc.1996.168

**Published:** 1996-04

**Authors:** N. H. Cox, T. C. Aitchison, J. M. Sirel, R. M. MacKie

**Affiliations:** Department of Dermatology, Cumberland Infirmary, Carlisle, UK.

## Abstract

A study of 953 invasive cutaneous malignant melanomas of the head and neck was performed to determine differences between lentigo maligna melanoma and other histogenetic types with regard to patients and sites affected; prognosis was analysed in 595 of these cases. The cases studied comprised all head and neck melanomas registered with the Scottish Melanoma Group between 1979 and 1992, apart from the 3% of cases that were unclassifiable or rare histogenetic types. The histogenetic types of melanoma were 498 (52%) lentigo maligna melanoma (LMM), 237 (25%) superficial spreading melanoma (SSM) and 218 (23%) nodular melanoma (NM). All types increased in incidence throughout the study period. Patients with LMM (mean age 73 years) and NM (mean 68 years) were significantly older than those with SSM (mean 57 years). There were significant anatomical subsite differences related to sex of patients and histogenetic type of melanoma; melanomas on the face were more frequent in females and 90% of LMM occurred at this site, whereas melanomas on the scalp, neck and ears were more frequent in men. Kaplan-Meier estimates of the probability of survival were produced for the 595 of these 953 patients with 5 year follow-up details. In this group of patients the prognostic significance of tumour thickness, Clark level of invasion, ulceration, histogenetic type of melanoma and number of mitoses were studied using stepwise variable selection of procedures. Each of these possible prognostic factors attained individual significance but the tumour thickness was the dominant risk factor in the proportional hazards analysis. When patients were divided into four sex/ulceration subgroups (male/ulcerated, female/ulcerated, male/non-ulcerated, female/non-ulcerated) and analysed by proportional hazards analysis, no variable other than the tumour thickness had any further prognostic effect. Histogenetic type did not remain an independent prognostic variable at this stage. Despite sex and subsite differences, the prognosis for invasive lentigo maligna melanoma does not differ from that for other histogenetic types after controlling for tumour thickness.


British Journal of Cancer (1996) 73, 940-944
© 1996 Stockton Press All rights reserved 0007-0920/96 $12.00

### Comparison between lentigo maligna melanoma and other histogenetic types of malignant melanoma of the head and neck

NH Cox[1], TC Aitchison[2], JM Sirel[2] and RM MacKie[3] for and on behalf of the Scottish Melanoma Group

[1]*Department of Dermatology, Cumberland Infirmary, Carlisle CA2 7HY;* [2]*Department of Statistics, University of Glasgow, Glasgow G12 8QW;* [3]*Department of Dermatology, University of Glasgow, Glasgow G12 8QQ, UK.*

**Summary** A study of 953 invasive cutaneous malignant melanomas of the head and neck was performed to determine differences between lentigo maligna melanoma and other histogenetic types with regard to patients and sites affected; prognosis was analysed in 595 of these cases. The cases studied comprised all head and neck melanomas registered with the Scottish Melanoma Group between 1979 and 1992, apart from the 3% of cases that were unclassifiable or rare histogenetic types. The histogenetic types of melanoma were 498 (52%) lentigo maligna melanoma (LMM), 237 (25%) superficial spreading melanoma (SSM) and 218 (23%) nodular melanoma (NM). All types increased in incidence throughout the study period. Patients with LMM (mean age 73 years) and NM (mean 68 years) were significantly older than those with SSM (mean 57 years). There were significant anatomical subsite differences related to sex of patients and histogenetic type of melanoma; melanomas on the face were more frequent in females and 90% of LMM occurred at this site, whereas melanomas on the scalp, neck and ears were more frequent in men. Kaplan–Meier estimates of the probability of survival were produced for the 595 of these 953 patients with 5 year follow-up details. In this group of patients the prognostic significance of tumour thickness, Clark level of invasion, ulceration, histogenetic type of melanoma and number of mitoses were studied using stepwise variable selection of procedures. Each of these possible prognostic factors attained individual significance but the tumour thickness was the dominant risk factor in the proportional hazards analysis. When patients were divided into four sex/ulceration subgroups (male/ulcerated, female/ulcerated, male/non-ulcerated, female/non-ulcerated) and analysed by proportional hazards analysis, no variable other than the tumour thickness had any further prognostic effect. Histogenetic type did not remain an independent prognostic variable at this stage. Despite sex and subsite differences, the prognosis for invasive lentigo maligna melanoma does not differ from that for other histogenetic types after controlling for tumour thickness.

**Keywords:** malignant melanoma; head and neck tumour

In Caucasians, about 10–20% of all malignant melanomas occur on the head and neck (McGovern *et al.*, 1980; Sober *et al.*, 1980; MacKie *et al.*, 1985; Cox *et al.*, 1987; Fisher, 1989; O'Brien *et al.*, 1991; Langford *et al.*, 1993), and therefore comprise a numerically important subset of a malignancy that has shown increasing incidence over several decades in most populations worldwide. Malignant melanoma of the head and neck has some notable differences from those at other body sites, especially a higher frequency of the lentigo maligna type of melanoma (McGovern *et al.*, 1980 ; Sober *et al.*, 1980; MacKie *et al.*, 1985; Cox *et al.*, 1987; Fisher, 1989; Langford *et al.*, 1993). From a management point of view, an important difference compared with other sites is that excision margins of thicker malignant melanomas of the head and neck may be limited by functional or cosmetic considerations.

Although tumour thickness (Breslow, 1970) at these, as at other sites, is accepted as a major prognostic factor, there is some variation between studies regarding other prognostic factors. One specific important question that remains is whether the prognosis for lentigo maligna melanoma differs significantly from the prognosis for melanoma of other histogenetic types. Many of the larger studies have originated from selected populations treated at tertiary referral centres in countries with high levels of sun exposure (Conley and Pack, 1963; Ballantyne, 1970; McGovern *et al.*, 1980; Gussack *et al.*, 1983; Urist *et al.*, 1984; Fisher, 1989; O'Brien *et al.*, 1991; Langford *et al.*, 1993). We have therefore studied the clinicopathological features of 953 malignant melanomas of the head and neck registered with the Scottish Melanoma Group (SMG) between 1979 and 1992 inclusive. These comprise all cases of head and neck melanoma diagnosed in Scotland over this 14 year period and are therefore a valuable database for population-based epidemiology. To clarify prognostic issues discussed in an earlier study (Cox *et al.*, 1987), we have analysed prognostic factors in the 595 cases in this database for whom follow-up data was available until at least the fifth year after diagnosis (all cases 1979–86).

Correspondence: NH Cox
Received 7 March 1995; revised 12 October 1995; accepted 27 October 1995

### Methods

Details of 953 patients with clinical stage I invasive malignant melanoma of the head and neck were available. These comprised the 97% of cases of invasive malignant melanoma of the three main histogenetic types at this body site that had been registered with the Scottish Melanoma Group from 1979 to 1992 (the other 3% were melanomas of unclassifiable histogenetic type); patients with non-invasive lentigo maligna or intraepidermal (Clark level I) *in situ* malignant melanoma were excluded. Registration methods have been described in detail elsewhere (MacKie *et al.*, 1985). The registrations, to the best of our knowledge, comprise all malignant melanomas occurring in the population (5 million persons) in Scotland, and are updated by contacting the responsible clinician periodically for details of outcome.

The factors that were considered comprised age and sex of patients, histogenetic type of melanoma, and anatomical subsite affected (face, scalp, neck, ears). Differences between subgroups involving these variables were analysed using Wilcoxon–Mann–Whitney, or $\chi^2$ tests as appropriate, and allowing for multiple comparisons by means of a Bonferroni-based procedure. Survival profiles were based on deaths due to melanoma; deaths due to unrelated causes were treated as censored data.

For the 595 patients diagnosed from 1979 to 1986, in whom follow-up details were complete to the fifth year after diagnosis, additional factors were analysed by univariate analysis for their prognostic significance. These were tumour thickness, Clark level of invasion, presence of ulceration and number of mitoses (low, medium, high). Other more detailed analyses were performed as described below; the numbers of cases entered into each of these is documented in the details of each analysis and was determined by the number with complete data for all relevant parts of the analysis in question. In 516 of these 595 patients (87%) there was complete information on all of the prognostic factors considered (Table III), as well as on the outcome in terms of deaths due to melanoma.

Kaplan–Meier estimates of the probability of survival were used to describe the survival profiles, and the effects of the potential prognostic risk factors were studied using log-rank tests and proportional hazards modelling, which involved forward stepwise and backward elimination variable selection procedures (Everitt, 1989). A previous study analysing the full SMG database for prognosis of melanoma at all body sites had demonstrated that the most important combinations of risk factors involved tumour thickness, ulceration and sex of patients (MacKie *et al.*, 1995). A separate stepwise proportional hazards analysis was therefore performed for each of the four sex/ulceration subgroups (male ulcerated, male non-ulcerated, female ulcerated, female non-ulcerated); the prognostic factors entered for this analysis were complete in 546 patients for whom a minimum of 5 year follow-up was available.

### Results

### *Incidence by histogenetic type*

All histogenetic types of malignant melanoma of the head and neck increased in incidence over the period studied (Figure 1). Malignant melanoma at this site accounted for 21% of the total number of registrations for all body sites; lentigo maligna melanoma (LMM) on the head and neck accounted for 83% of all cases of LMM. The proportions of the different histogenetic types (see Table I for absolute values) were: LMM 52%, superficial spreading melanoma (SSM) 25%, nodular melanoma (NM) 23%.

### *Age and sex related to histogenetic type*

There were significant differences among histogenetic types relating to the age of patients (Table I). Patients with LMM or with NM were significantly older than those with SSM (both $P<0.0001$, Wilcoxon–Mann–Whitney test); those with LMM were older than those with NM but this was not statistically significant. The ratio of females to males for each histogenetic type was LMM 1.74:1, SSM 1.24:1, NM 0.98:1 (Table I). For each of these histogenetic types, the mean age for female patients was 2–3 years greater than that for males (not statistically significant).

**Figure 1** Registrations of melanoma of the head and neck in Scotland 1979–92. (——), Total; (......), LMM; (- - - -), SSM; ( – – – ), NM.

### *Anatomical subsite*

The anatomical subsite distribution of malignant melanoma on the head and neck is shown in Table II, related to sex of patients and to histogenetic type of melanoma. All histogenetic types were most frequent on the face but there were significant differences in the number of each type at this site (which accounted for 90% of LMM but only 70% of NM and 56% of SSM, ($P<0.00001$, $\chi^2$ test). There were also significant differences in the distribution of histogenetic types on the neck ($P<0.0001$, $\chi^2$ test) and ears ($P<0.05$, $\chi^2$ test). There were only four cases of malignant melanoma of the lip, which are not included in the Table.

There were significant differences between the sexes in the proportions of melanoma on the face ($P<0.0001$, $\chi^2$ test), scalp ($P<0.001$, $\chi^2$ test) and ears ($P<0.001$, $\chi^2$ test). Specifically, females have the great majority of melanomas on the face whereas males have a significantly higher proportion on the neck and ears.

### *Prognosis*

A Kaplan–Meier estimate of the survival profile produced for all patients demonstrated a steady slow decrease in survival over a 10 year period, the 5 year and 10 year survival figures being 85% and 75% respectively. The same analysis for each histogenetic type separately demonstrated better survival for the LMM histogenetic subtype when compared with the other subtypes in isolation, but 63% of these were less than 1.5 mm thick and therefore expected to have a good prognosis. As will be seen below, histogenetic type did not remain an independent prognostic factor when a stepwise proportional hazards model was used.

The individual significance of each of the possible prognostic risk factors was statistically assessed (Table III), demonstrating that prognosis was significantly related to each of the following in isolation: sex of patients, histogenetic type of melanoma, presence of ulceration, tumour thickness, Clark level of invasion and level of mitotic activity. A stepwise proportional hazards analysis was performed, which demonstrated (Table III) that tumour thickness was clearly the dominant risk factor. Male sex, ulceration and deep invasion (level 5) all had a significant adverse effect after controlling for thickness and for each other. LMM had a better prognosis when compared with NM and SSM, but this was eliminated after controlling for thickness and the other significant variables.

Since the two categorical factors, sex and presence/absence of ulceration, were the two most important risk factors after correcting for tumour thickness, and since a similar approach proved effective in analysis of the whole of the SMG database (MacKie *et al.*, 1995), a separate stepwise proportional hazards analysis was performed for each of the four sex-ulceration subgroups (i.e. females with ulcerated lesions, females with non-ulcerated lesions, males with ulcerated lesions, and males with non-ulcerated lesions). Once divided into these groups, only the tumour thickness had any further prognostic effect, i.e. the interactions between sex, ulceration and tumour thickness eliminated the effect of

**Table I** Numbers of head and neck malignant melanoma studied, demonstrating age and sex differences between histogenetic types.

| *Histogenetic type* | *Number (%)* Females | Males | Total | *Age (years)* Mean | Median | Range |
|---|---|---|---|---|---|---|
| LMM | 316 (57%) | 182 (46%) | 498 (52%) | 73 | 74 | 27–97[a] |
| SSM | 131 (24%) | 106 (27%) | 237 (25%) | 57 | 60 | 15–99 |
| NM | 108 (19%) | 110 (28%) | 218 (23%) | 68 | 73 | 15–100[b] |
| Total | 555 | 398 | 953 | | | |

[a] LMM average age significantly older than SSM, $P<0.0001$. [b] NM average age significantly older than SSM, $P<0.0001$.

Table II Distribution of malignant melanoma by anatomical site, related to sex of patients and histogenetic type of melanoma[a]

| | *Sex of patients* | | *Histogenetic type of melanoma* | | |
|---|---|---|---|---|---|
| *Site* | *Female* | *Male* | *LMM* | *SSM* | *NM* |
| Face | 473 (86%) | 255 (64%) | 445 (90%) | 131 (56%) | 152 (70%) |
| Scalp | 10 (2%) | 34 (9%) | 16 (3%) | 14 (6%) | 14 (6%) |
| Neck | 52 (9%) | 67 (17%) | 18 (4%) | 69 (29%) | 32 (15%) |
| Ears | 16 (3%) | 42 (11%) | 18 (4%) | 22 (9%) | 18 (8%) |

[a] Four patients with lip lesions not included.

Table III Results of survival analyses on factors affecting melanoma prognosis

| | *Individual significance on full survival data* | | *Order of inclusion in stepwise proportional hazards analysis* | | |
|---|---|---|---|---|---|
| *Factor* | $\chi^2$ (d.f.) | P-*value* | *Order* | $\chi^2$ (d.f.) | P-*value for inclusion* |
| Tumour thickness | 83 (1) | <0.0001 | 1 | 83 (1) | <0.0001 |
| Level of invasion (2,3,4,5) | 50 (3) | <0.0001 | 4 | 6 (1)[a] | 0.02 |
| Ulceration (yes, no) | 46 (1) | <0.0001 | 3 | 6 (1) | 0.02 |
| Level of mitosis (low, medium, high) | 18 (2) | <0.001 | | Not included[b] | |
| Histogenetic type (LMM, non-LMM) | 20 (1) | <0.001 | | Not included[b] | |
| Sex (female, male) | 10 (1) | <0.005 | 2 | 9 (1) | <0.005 |
| Site (face, scalp, neck, ears) | 5 (3) | 0.17 | | Not included[b] | |
| Age | 0.45 (1) | 0.50 | | Not included[b] | |

[a] Prognosis for level of invasion 5 is significantly worse than *all* other levels which show no significant differences in prognosis with respect to each other. [b] This factor did not achieve siginficance (i.e. *P*-value>0.05) when tested in a proportional hazards model including the four significant factors (i.e. tumour thickness, sex, ulceration and level of invasion).

level of invasion demonstrated in the simpler stepwise analysis. The survival profiles for the four sex/ulceration subgroups clearly demonstrated that increasing tumour thickness reduced survival prospects in all four groups, the worst prognosis being in men with thick ulcerated melanomas.

### Discussion

This study is one of the largest analyses of invasive head and neck melanoma reported, and is the most complete study based on a geographically defined population rather than an institutional series. It is also notable in covering patients living in a temperate climate; with few exceptions (Ringborg *et al.*, 1993; Andersson *et al.*, 1993) other studies with a similar number of cases have been in areas of the world with much greater sunlight exposure. This may explain some of the differences in histogenetic types and anatomical sites of melanoma between the present study and other reported series.

Despite having excluded lentigo maligna/LMM *in situ*, which mainly occur on the face, and all other *in situ* melanomas, the proportion of malignant melanomas arising on the head and neck in the present study (21% of the total Scottish Melanoma Group database) is higher than the 11–17% reported in other large studies (Sober *et al.*, 1980; Gussack *et al.*, 1983; Fisher, 1989; O'Brien *et al.*, 1991; Langford *et al.*, 1993). Most striking is the high proportion of patients with LMM (52%), which is considerably greater than the 24% reported in a recent large European study (Ringborg *et al.*, 1993), and 15–16% in large Australian and American studies (Fisher, 1989; O'Brien *et al.*, 1991; Langford *et al.*, 1993). These head and neck LMM accounted for 83% of all LMM registered with the SMG, a similar proportion to that reported by many authors (Conley and Pack, 1963; Sober *et al.*, 1980; Blois *et al.*, 1983), although the reported proportion varies from 36% (Van der Esch, cited by McGovern *et al.*, 1980) to 92% (McGovern *et al.*, 1980). The older age of patients with LMM in the present and other studies (McGovern *et al.*, 1980; Holman and Armstrong, 1984; Langford *et al.*, 1993; Ringborg *et al.*, 1993), is consistent with the theory that LMM is related more to cumulative lifetime sunlight exposure than the other varieties, which are postulated to be related to acute intermittent sunlight exposure (MacKie, 1981; Holman *et al.*, 1983; Swerdlow, 1984; Elwood *et al.*, 1987). Depending on occupation and leisure interests, the head and neck may be exposed to sunlight in either of these patterns. However, the steady increase in all three main histogenetic subtypes of melanoma during the study period is not in support of different aetiologies (the sharp increase in SMM in 1985, shown in Figure 1, is probably due to a Scottish melanoma educational campaign as registrations increased at all body sites in this year). Furthermore, personal factors identified as risk factors for lentigo maligna and lentigo maligna melanoma in a recent study (McHenry *et al.*, 1995) are also recognised as risk factors for SSM and NM.

The results of the present study confirm our earlier report (Cox *et al.*, 1987) of significant anatomical subsite differences between the sexes and for histogenetic types of melanoma. The overall distribution of melanoma at different subsites (face, scalp, ear and neck) is similar to other large series (Fisher, 1989; O'Brien *et al.*, 1991; Ringborg *et al.*, 1993), and the male predominance of melanoma on the neck, scalp and ears also confirms results of other authors (Fitzpatrick *et al.*, 1972; Knutson *et al.*, 1972; Byers *et al.*, 1980; Day *et al.*, 1982*a*; Gussack *et al.*, 1983; Fisher, 1989; O'Brien *et al.*, 1991; Ringborg *et al.*, 1993). The reasons for this are uncertain but baldness and shorter hair styles in men are likely to be the explanation. Most studies of head and neck melanoma have reported a male predominance (Kragh and

Erich, 1960; Conley and Pack, 1963; Catlin, 1966; Ballantyne, 1970; Fitzpatrick *et al.*, 1972; Knutson *et al.*, 1972; Donellan *et al.*, 1972; Simons, 1972; Ames *et al.*, 1976; Gussack *et al.*, 1983; Urist *et al.*, 1984; Fisher, 1989; O'Brien *et al.*, 1991), although a few studies (McGovern *et al.*, 1980; Wanebo *et al.*, 1988; Ringborg *et al.*, 1993; Andersson *et al.*, 1993) support our finding an excess of females. Where data for other body sites is available from the same study population, the male–female ratio is higher for the head and neck than for other body sites (Hansen and McCarten, 1974; Ames *et al.*, 1976; Gussack *et al.*, 1983; Cox *et al.*, 1987; Langford *et al.*, 1993) and an absolute or relative excess of females with LMM is generally reported in studies where this has been analysed (McGovern *et al.*, 1980; Cox *et al.*, 1987; Ringborg *et al.*, 1993).

It is interesting to consider reasons for the differences between the Swedish Melanoma Group study (Ringborg *et al.*, 1993) and the present report from a similar latitude. LMM was less frequent in that study (24% compared with our 52%), although the anatomical subsite and sex differences were similar to our results. In particular, LMM in the Swedish study was overrepresented in females (female–male ratio 2.4:1 compared with 1.1:1 for all sites in their study population), and the LMM prognosis was better. However, it is not stated whether the Swedish data include non-invasive LMM, which would clearly improve the prognosis for the LMM group, and unfortunately the authors felt that LMM was a separate entity and excluded it from the detailed analysis of survival.

Potential prognostic factors for melanoma that were analysed in the present study were those identified in large studies of melanoma at any site. Because there are many prognostic variables, which may all have individual significance, but which may be interrelated (e.g. thickness and depth of invasion), the stepwise analysis was used to identify the most important prognostic indicators. This demonstrated that male sex and presence of ulceration significantly worsened survival but confirmed the tumour thickness to be the most important factor, as determined in virtually all studies of melanoma. The survival profiles for the four sex/ulceration subgroups clearly demonstrated that increasing tumour thickness reduced survival prospects in all four groups. Ulceration is a significant predictive factor in many studies that have controlled for tumour thickness (Balch *et al.*, 1978, 1980; Van der Esch, 1981; Day *et al.*, 1982*b*; Urist *et al.*, 1984; Shaw *et al.*, 1985; O'Brien *et al.*, 1991; Andersson *et al.*, 1993; Langford *et al.*, 1993; MacKie *et al.*, 1995) and even retains significance in patients with lymph node metastasis (Balch *et al.*, 1980). Male sex has been associated with poorer prognosis in most studies of head and neck melanoma (Ballantyne, 1970; Fitzpatrick *et al.*, 1972; Hansen and McCarten, 1974; Gussack *et al.*, 1983; Cox *et al.*, 1987; O'Brien *et al.*, 1991; Langford *et al.*, 1993; Ringborg *et al.*, 1993; Andersson *et al.*, 1993), although some studies report no sex difference (Catlin, 1966; Knutson *et al.*, 1972; McGovern *et al.*, 1980) and one reported a better prognosis in men (Southwick *et al.*, 1963).

Several authors have identified melanoma of the head and neck as having a worse prognosis than other body sites (Day *et al.*, 1982*b*; Blois *et al.*, 1983; Gussack *et al.*, 1983; Urist *et al.*, 1984; Fisher, 1989; Hersey *et al.*, 1991), and some have identified subsite differences in prognosis for head and neck melanomas on univariate (Ballantyne, 1970) or multivariate (Urist *et al.*, 1984; Wanebo *et al.*, 1988; Fisher, 1989; O'Brien *et al.*, 1991; Ringborg *et al.*, 1993) analysis. In general, a worse prognosis has been reported for scalp (Urist *et al.*, 1984; Wanebo *et al.*, 1988; Fisher, 1989; O'Brien *et al.*, 1991; Ringborg *et al.*, 1993) or scalp and ear (Wanebo *et al.*, 1988). Despite the differences in sex of patients and histogenetic types of melanoma at different anatomical subsites, the site of melanoma did not have independent significance in the proportional hazards analysis in the present study.

LMM has been reported to have a better prognosis than other histogenetic types of melanoma, but this data is confounded both by inclusion of lentigo maligna (McGovern *et al.*, 1980) and by failure to control for tumour thickness in older studies. Some studies using multivariate analyses do still report a better prognosis for this histogenetic type of melanoma (Urist *et al.*, 1984; Wanebo *et al.*, 1988; O'Brien *et al.*, 1991). However, other authors are in agreement with our conclusion that, thickness for thickness, LMM does not have a better prognosis than other types of melanoma once invasion has occurred and when sex and ulceration are taken into account (Gussack *et al.*, 1983; Koh *et al.*, 1984; Pittelkow *et al.*, 1986; Langford *et al.*, 1993). The report by Urist *et al.* (1984), which indicated that ulceration was a more important variable than thickness for LMM, is not supported by the present or most other studies, although ulceration is undoubtedly an important prognostic feature after accounting for tumour thickness. Our results clearly demonstrate that the most important prognostic variable for all histogenetic types of head and neck melanoma is the tumour thickness.

**Acknowledgements**
We are grateful to the Cancer Research Campaign and the Scottish Home and Health Department, who fund the Scottish Melanoma Group, to the Scottish Hospitals Endowment Research Trust, who funded this investigation, and to Evelyn Salt and Jenny Stewart for their assistance in accessing the database.

### References

AMES FC, SUGARBAKER EV AND BALLANTYNE AJ. (1976). Analysis of survival and disease control in stage I melanoma of the head and neck. *Am. J. Surg.*, **132,** 484–491.

ANDERSSON AP, GOTTLIEB J, SONDERGAARD K, HOU-JENSEN K AND DRZWIECKI KT. (1993). Significant prognostic factors for recurrence-free survival after surgical treatment of head-neck melanoma. *Ugeskrift for Laeger*, **155,** 2397–2399.

BALCH CM, MURAD TM, SOONG S-J, INGALLS AL, HALPERN NB AND MADDOX WA. (1978). A multifactorial analysis of melanoma: prognostic histopathological features comparing Clark's and Breslow's staging methods. *Ann. Surg.*, **188,** 732–742.

BALCH CM, WILKERSON JA, MURAD TM, SOONG S-J, INGALLS AL AND MADDOX WA. (1980). The prognostic significance of ulceration of cutaneous melanoma. *Cancer*, **45,** 3012–3017.

BALLANTYNE AJ. (1970). Malignant melanoma of the skin of the head and neck. *Am. J. Surg.*, **120,** 425–431.

BLOIS MS, SAGEBIEL RW, ABARBANEL RM, CALDWELL TM AND TUTTLE MS. (1983). Malignant melanoma of the skin. 1. The association of tumour depth and type, and patient sex, age, and site with survival. *Cancer*, **52,** 1330–1341.

BRESLOW A. (1970). Thickness, cross-sectional area and depth of invasion in the prognosis of cutaneous melanoma. *Ann. Surg.*, **172,** 902–908.

BYERS RM, SMITH JL, RUSSELL N AND ROSENBERG V. (1980). Malignant melanoma of the external ear. Review of 102 cases. *Am. J. Surg.*, **140,** 518–521.

CATLIN D. (1966). Cutaneous melanoma of the head and neck. *Am. J. Surg.*, **112,** 512–521.

CONLEY JJ AND PACK GT. (1963). Melanoma of head and neck. *Surg. Gynecol. Obstet.*, **116,** 15–28.

COX NH, JONES SK AND MACKIE RM. (1987). Malignant melanoma of the head and neck in Scotland: an eight-year analysis of trends in prevalence, distribution and prognosis. *Quart. J. Med., New Series*, **64,** 661–670.

DAY Jr CL, MIHM Jr MC, LEW RA, HARRIS MN, KOPF AW, FITZPATRICK TB, HARRIST TJ, GOLOMB FM, POSTEL A, HENNESSEY P, GUMPORT SL, RAKER JW, MALT RA, COSIMI AB, WOOD WC, ROSES DF, GORSTEIN F, RIGEL D, FRIEDMAN RJ, MINTZIS MM AND SOBER AJ. (1982*a*). Prognostic factors for melanoma patients with lesions 0.76-1.69 mm in thickness. *Ann. Surg.*, **195,** 30–34.

DAY Jr CL, MIHM Jr MC, LEW RA, HARRIS MN, KOPF AW, FITZPATRICK TB, HARRIST TJ, GOLOMB FM, POSTEL A, HENNESSEY P, GUMPORT SL, RAKER JW, MALT RA, COSIMI AB, WOOD WC, ROSES DF, GORSTEIN F, RIGEL D, FRIEDMAN RJ, MINTZIS MM AND SOBER AJ. (1982*b*). Prognostic factors for patients with clinical stage 1 melanoma of intermediate thickness (1.51-3.99 mm). A conceptual model for tumor growth and metastasis. *Ann. Surg.*, **195**, 35–43.

DONELLAN MJ, SEEMAYER T, HUVOS AG, MIKE V AND STRONG EW. (1972). Clinicopathologic study of cutaneous melanoma of the head and neck. *Am. J. Surg.*, **124**, 450–455.

ELWOOD JM, GALLAGHER RP, WORTH AJ, WOOD WS AND PEARSON JC. (1987). Etiological differences between subtypes of cutaneous malignant melanoma: Western Canada Melanoma study. *J. Natl Cancer Inst.*, **78**, 37–44.

EVERITT BS. (1989). *Statistical Methods for Medical Investigations*. pp. 83–98. Oxford University Press: Oxford.

FISHER SR. (1989). Cutaneous malignant melanoma of the head and neck. *Laryngoscope*, **99**, 822–836.

FITZPATRICK PJ, BROWN TC AND REID J. (1972). Malignant melanoma of the head and neck: a clinicopathological study. *Can. J. Surg.*, **15**, 90–100.

GUSSACK GS, REINTGEN D, COX E, FISHER SR, COLE TB AND SEIGLER HF. (1983). Cutaneous melanoma of the head and neck. *Arch. Otolaryngol*, **109**, 803–808.

HANSEN MG AND MCCARTEN AB. (1974). Tumor thickness and lymphocytic infiltration in malignant melanoma of the head and neck. *Am. J. Surg.*, **128**, 557–561.

HERSEY P, SILLAR RW, HOWE CG, BURTON RC, DARBAR SV, FOSTER HM, COLLINS SM, BRADLEY DE AND OWENS D. (1991). Factors related to the presentation of patients with thick primary melanomas. *Med. J. Austral.*, **154**, 583–587.

HOLMAN CDJ AND ARMSTRONG BK. (1984). Cutaneous malignant melanoma and indicators of total accumulated exposure to the sun: an analysis separating histogenetic types. *J. Natl Cancer Inst.*, **73**, 75–82.

HOLMAN CDJ, ARMSTRONG BK AND HEENAN PJ. (1983). A theory of the etiology and pathogenesis of human cutaneous malignant melanoma. *J. Natl Cancer Inst.*, **71**, 651–656.

KNUTSON CO, HORI JM AND WATSON FR. (1972). Melanoma of the head and neck. *Am. J. Surg.*, **124**, 542–553.

KOH HK, MICHALIK E, SOBER AJ, LEW RA, DAY CL, CLARK W, MIHM MC, KOPF AW, BLOIS MS AND FITZPATRICK TB. (1984). Lentigo maligna melanoma has no better prognosis than other types of melanoma. *J. Clin. Oncol.*, **2**, 994–1001.

KRAGH LV AND ERICH JB. (1960). Malignant melanomas of the head and neck. *Ann. Surg.*, **151**, 91–96.

LANGFORD FPJ, FISHER SR, MOLTER DW AND SEIGLER HF. (1993). Lentigo maligna melanoma of the head and neck. *Laryngoscope*, **103**, 520–524.

MCGOVERN VJ, SHAW HM, MILTON GW AND FARAGO GA. (1980). Is malignant melanoma arising in a Hutchinson's freckle a separate disease entity? *Histopathology*, **4**, 235–242.

MCHENRY PM, AITCHISON T AND MACKIE RM. (1995). Risk factors for lentigo maligna/lentigo maligna melanoma. *Br. J. Dermatol.*, **133** (Suppl 45). 23.

MACKIE RM. (1981). The role of sunlight in the aetiology of cutaneous malignant melanoma. *Clin. Exp. Dermatol.*, **6**, 407–410.

MACKIE RM, SMYTH JF, SOUTAR DS, CALMAN KC, WATSON ACH, HUNTER JAA, MCLAREN KM, MACGILLIVRAY JB, MCPHIE JL, RANKIN R, HUTCHEON AW AND KEMP IW. (1985). Malignant melanoma in Scotland 1979–1985. *Lancet*, **2**, 859–862.

MACKIE RM, AITCHISON T, SIREL J, MCLAREN K AND WATT D. (1995). Prognostic models for subgroups of melanoma patients from the Scottish Melanoma Group database 1979–86, and their subsequent validation. *Br. J. Cancer*, **71**, 173–176.

O'BRIEN CJ, COATES AS, PETERSEN-SCHAEFER K, SHANNON K, THOMPSON JF, MILTON GW AND MCCARTHY WH. (1991). Experience with 998 cutaneous melanomas of the head and neck over 30 years. *Am. J. Surg.*, **162**, 310–314.

PITTELKOW MR, SU WPD, WICK M, SANCHEZ N AND PALESTINE R. (1986). Clinicopathologic study of lentigo maligna melanoma. *J. Cutan. Pathol.*, **13**, 75.

RINGBORG U, AFZELIUS L-E, LAGERLÔF B, ADAMI HO, AUGUSTSSON I, BLOMQVIST E, BOERYD B, CARLIN E, EDSTRÖM S, ELDH J, HANNER P, HANSSON J, JOHANSSON H, LINDHOLM C, MALEC E, NÄSLUND L, SCHNÜRER LB, SKÖLD S AND WERSÄLL J. (1993). Cutaneous malignant melanoma of the head and neck. Analysis of treatment results and prognostic factors in 581 patients: a report from the Swedish Melanoma Study Group. *Cancer*, **71**, 751–758.

SHAW HM, BALCH CM, SOONG S-J, MILTON GW AND MCCARTHY WH. (1985). Prognostic histopathological factors in malignant melanoma. *Pathology*, **17**, 271–274.

SIMONS JN. (1972). Malignant melanoma of the head and neck. *Am. J. Surg.*, **124**, 485–488.

SOBER AJ, FITZPATRICK TB AND MIHM JR MC. (1980). Primary melanoma of the skin: recognition and management. *J. Am. Acad. Dermatol.*, **2**, 179–197.

SOUTHWICK HW, SLAUGHTER DP AND HINKAMP JF. (1963). Malignant melanomas of the skin of the head and neck. *Am. J. Surg.*, **106**, 852–855.

SWERDLOW AJ. (1984). Epidemiology of cutaneous malignant melanoma. In: MacKIE RM (ed). *Clinics Oncol.*, **3**, 407–437.

URIST MM, BALCH CM, SOONG SJ, MILTON GW, SHAW HM, MCGOVERN VJ, MURAD TM, MCCARTHY WH AND MADDOX WA. (1984). Head and neck melanomas in 534 clinical stage I patients. A prognostic factors analysis and results of surgical treatment. *Ann. Surg.*, **200**, 769–775.

VAN DER ESCH EP, CASCINELLI N, PREDA F, MORABITO A AND BUFALINO R. (1981). Stage I melanoma of the skin: evaluation of prognosis according to histologic characteristics. *Cancer*, **48**, 1668–1673.

WANEBO HJ, COOPER PH, YOUNG DV, HARPOLE DH AND KAISER DL. (1988). Prognostic factors in head and neck melanoma. Effect of lesion location. *Cancer*, **62**, 831–837.


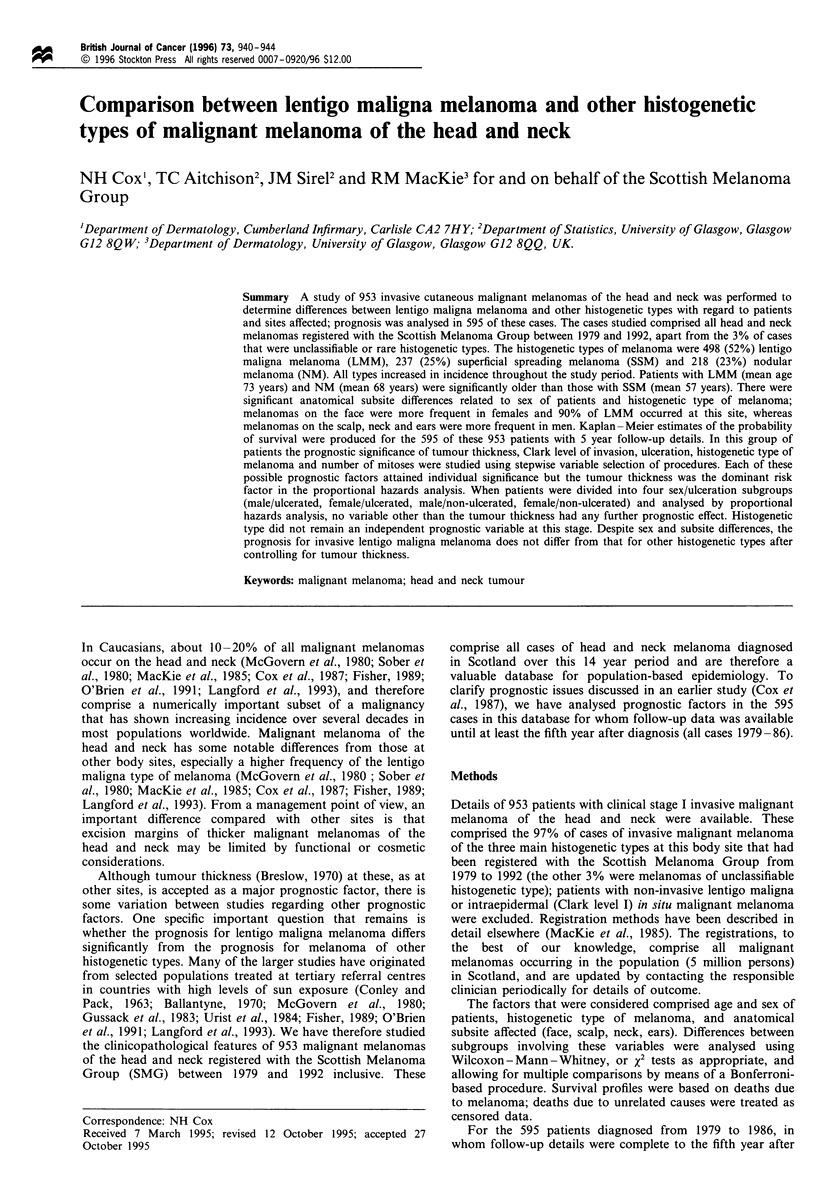

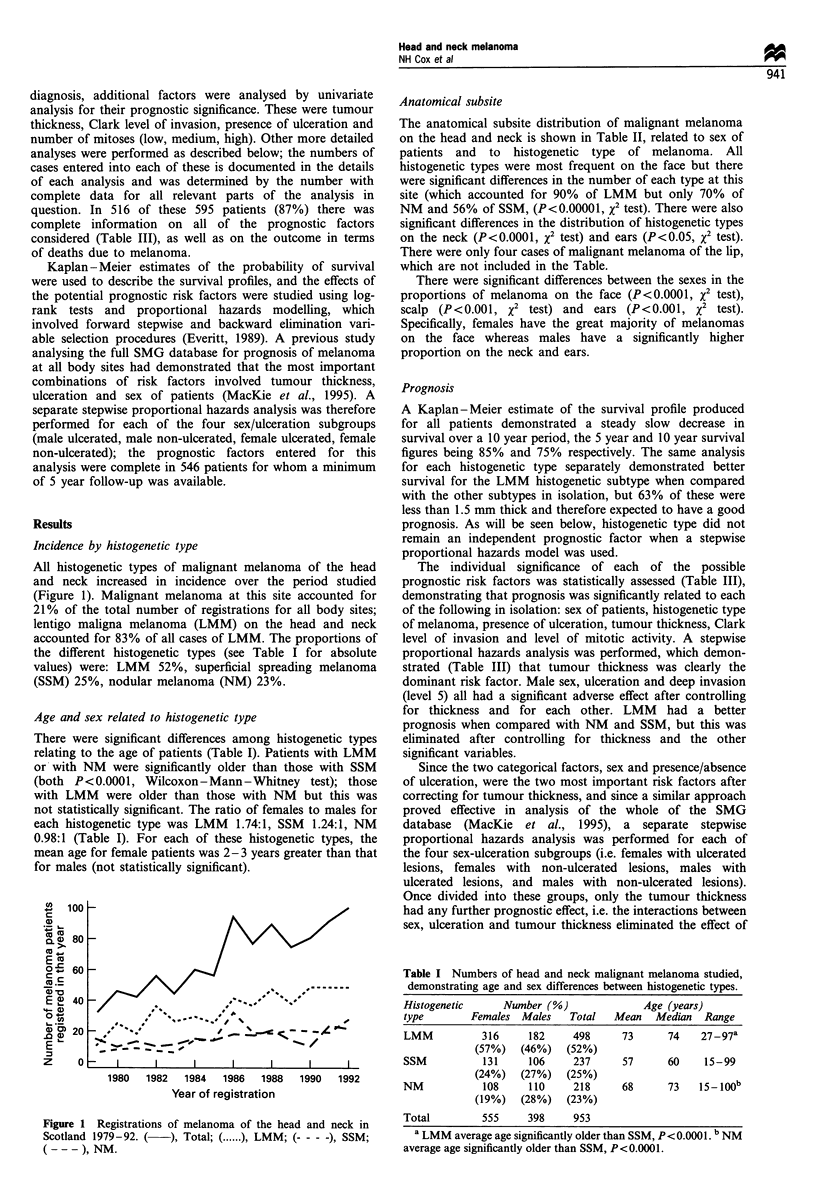

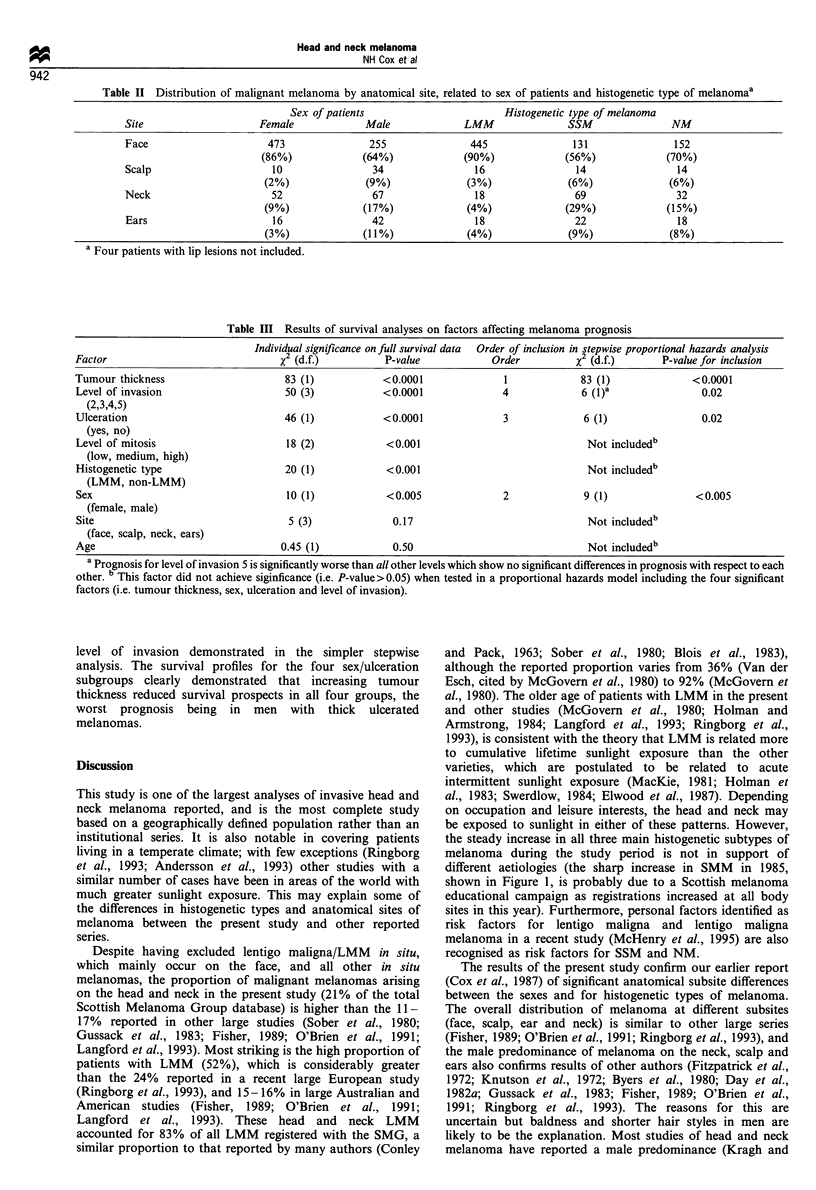

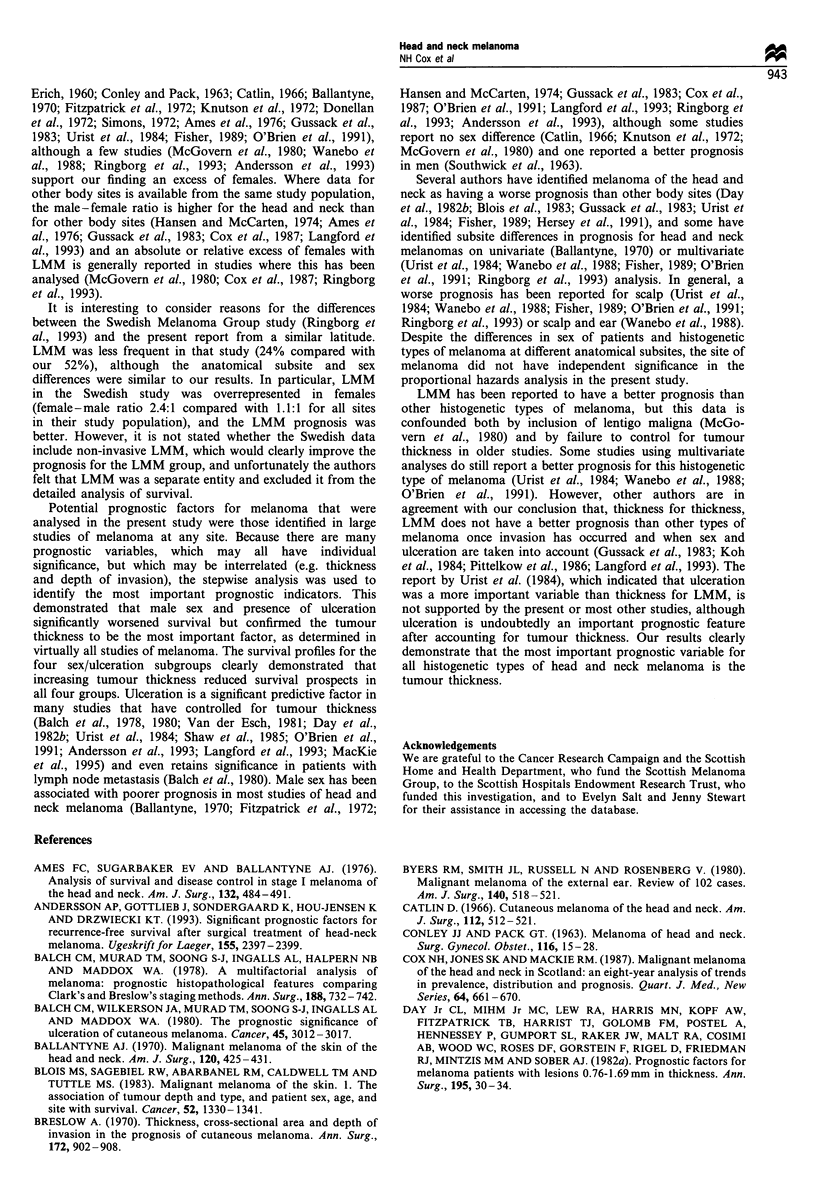

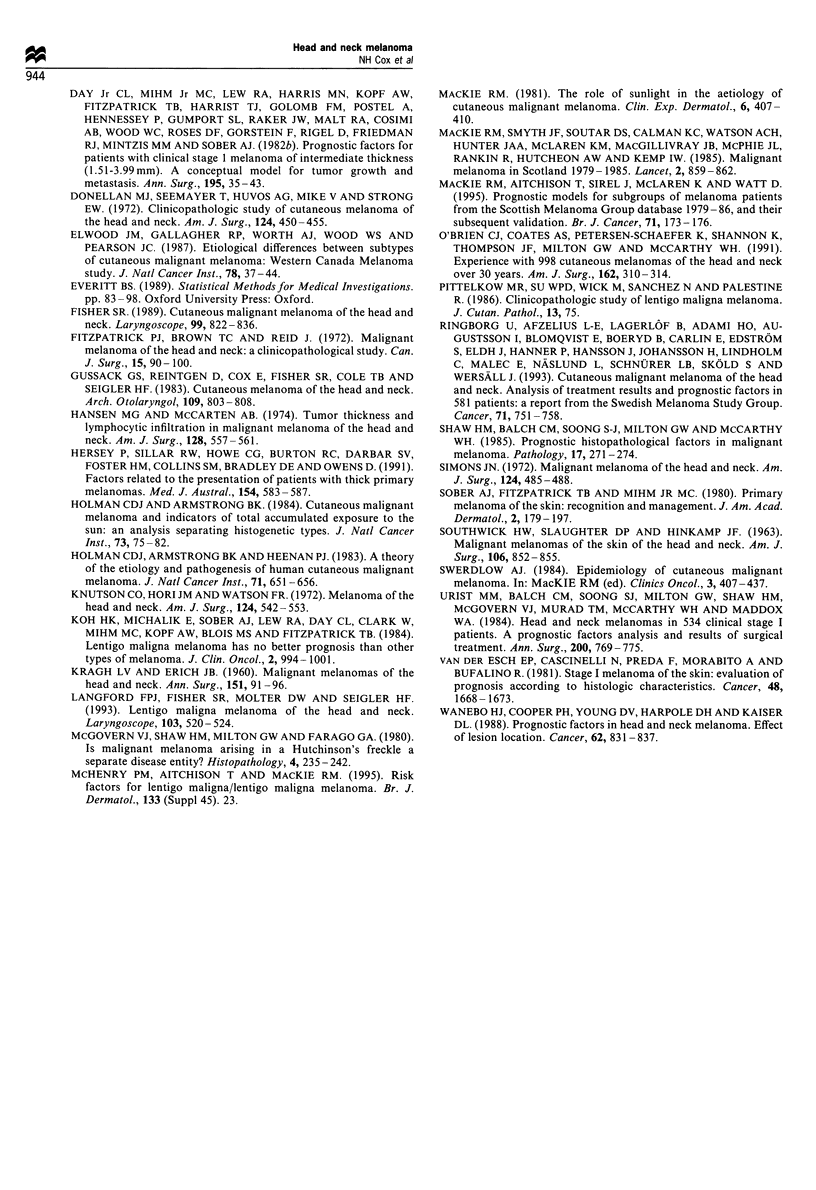


## References

[OCR_00566] Ames F. C., Sugarbaker E. V., Ballantyne A. J. (1976). Analysis of survival and disease control in stage I melanoma of the head and neck.. Am J Surg.

[OCR_00569] Andersson A. P., Gottlieb J., Søndergaard K., Hou-Jensen K., Drzewiecki K. T. (1993). Prognostiske faktorer af betydning for den recidivfrie overlevelse efter kirurgisk behandling af hoved-hals-melanom.. Ugeskr Laeger.

[OCR_00575] Balch C. M., Murad T. M., Soong S. J., Ingalls A. L., Halpern N. B., Maddox W. A. (1978). A multifactorial analysis of melanoma: prognostic histopathological features comparing Clark's and Breslow's staging methods.. Ann Surg.

[OCR_00583] Balch C. M., Wilkerson J. A., Murad T. M., Soong S. J., Ingalls A. L., Maddox W. A. (1980). The prognostic significance of ulceration of cutaneous melanoma.. Cancer.

[OCR_00587] Ballantyne A. J. (1970). Malignant melanoma of the skin of the head and neck. An analysis of 405 cases.. Am J Surg.

[OCR_00589] Blois M. S., Sagebiel R. W., Abarbanel R. M., Caldwell T. M., Tuttle M. S. (1983). Malignant melanoma of the skin. I. The association of tumor depth and type, and patient sex, age, and site with survival.. Cancer.

[OCR_00595] Breslow A. (1970). Thickness, cross-sectional areas and depth of invasion in the prognosis of cutaneous melanoma.. Ann Surg.

[OCR_00602] Byers R. M., Smith J. L., Russell N., Rosenberg V. (1980). Malignant melanoma of the external ear. Review of 102 cases.. Am J Surg.

[OCR_00611] CONLEY J. J., PACK G. T. (1963). Melanoma of the head and neck.. Surg Gynecol Obstet.

[OCR_00605] Catlin D. (1966). Cutaneous melanoma of the head and neck.. Am J Surg.

[OCR_00615] Cox N. H., Jones S. K., MacKie R. M. (1987). Malignant melanoma of the head and neck in Scotland: an eight-year analysis of trends in prevalence, distribution and prognosis.. Q J Med.

[OCR_00637] Day C. L., Mihm M. C., Lew R. A., Harris M. N., Kopf A. W., Fitzpatrick T. B., Harrist T. J., Golomb F. M., Postel A., Hennessey P. (1982). Prognostic factors for patients with clinical stage I melanoma of intermediate thickness (1.51 - 3.39 mm). A conceptual model for tumor growth and metastasis.. Ann Surg.

[OCR_00628] Day C. L., Mihm M. C., Sober A. J., Harris M. N., Kopf A. W., Fitzpatrick T. B., Lew R. A., Harrist T. J., Golomb F. M., Postel A. (1982). Prognostic factors for melanoma patients with lesions 0.76 - 1.69 mm in thickness. An appraisal of "thin" level IV lesions.. Ann Surg.

[OCR_00645] Donnellan M. J., Seemayer T., Huvos A. G., Miké V., Strong E. W. (1972). Clinicopathologic study of cutaneous melanoma of the head and neck.. Am J Surg.

[OCR_00647] Elwood J. M., Gallagher R. P., Worth A. J., Wood W. S., Pearson J. C. (1987). Etiological differences between subtypes of cutaneous malignant melanoma: Western Canada Melanoma Study.. J Natl Cancer Inst.

[OCR_00657] Fisher S. R. (1989). Cutaneous malignant melanoma of the head and neck.. Laryngoscope.

[OCR_00666] Gussack G. S., Reintgen D., Cox E., Fisher S. R., Cole T. B., Seigler H. F. (1983). Cutaneous melanoma of the head and neck. A review of 399 cases.. Arch Otolaryngol.

[OCR_00673] Hansen M. G., McCarten A. B. (1974). Tumor thickness and lymphocytic infiltration in malignant melanoma of the head and neck.. Am J Surg.

[OCR_00679] Hersey P., Sillar R. W., Howe C. G., Burton R. C., Darbar S. V., Foster H. M., Collins S. M., Bradley D. E., Owens D. (1991). Factors related to the presentation of patients with thick primary melanomas.. Med J Aust.

[OCR_00684] Holman C. D., Armstrong B. K. (1984). Cutaneous malignant melanoma and indicators of total accumulated exposure to the sun: an analysis separating histogenetic types.. J Natl Cancer Inst.

[OCR_00690] Holman C. D., Armstrong B. K., Heenan P. J. (1983). A theory of the etiology and pathogenesis of human cutaneous malignant melanoma.. J Natl Cancer Inst.

[OCR_00703] KRAGH L. V., ERICH J. B. (1960). Malignant melanomas of the head and neck.. Ann Surg.

[OCR_00700] Koh H. K., Michalik E., Sober A. J., Lew R. A., Day C. L., Clark W., Mihm M. C., Kopf A. W., Blois M. S., Fitzpatrick T. B. (1984). Lentigo maligna melanoma has no better prognosis than other types of melanoma.. J Clin Oncol.

[OCR_00709] Langford F. P., Fisher S. R., Molter D. W., Seigler H. F. (1993). Lentigo maligna melanoma of the head and neck.. Laryngoscope.

[OCR_00735] MacKie R. M., Aitchison T., Sirel J. M., McLaren K., Watt D. C. (1995). Prognostic models for subgroups of melanoma patients from the Scottish Melanoma Group database 1979-86, and their subsequent validation.. Br J Cancer.

[OCR_00729] MacKie R. M., Smyth J. F., Soutar D. S., Calman K. C., Watson A. C., Hunter J. A., McLaren K. M., MacGillivray J. B., McPhie J. L., Rankin R. (1985). Malignant melanoma in Scotland 1979-1983.. Lancet.

[OCR_00724] Mackie R. M. (1981). The role of sunlight in the aetiology of cutaneous malignant melanoma.. Clin Exp Dermatol.

[OCR_00714] McGovern V. J., Shaw H. M., Milton G. W., Farago G. A. (1980). Is malignant melanoma arising in a Hutchinson's melanotic freckle a separate disease entity?. Histopathology.

[OCR_00742] O'Brien C. J., Coates A. S., Petersen-Schaefer K., Shannon K., Thompson J. F., Milton G. W., McCarthy W. H. (1991). Experience with 998 cutaneous melanomas of the head and neck over 30 years.. Am J Surg.

[OCR_00753] Ringborg U., Afzelius L. E., Lagerlöf B., Adami H. O., Augustsson I., Blomqvist E., Boeryd B., Carlin E., Edström S., Eldh J. (1993). Cutaneous malignant melanoma of the head and neck. Analysis of treatment results and prognostic factors in 581 patients: a report from the Swedish Melanoma Study Group.. Cancer.

[OCR_00774] SOUTHWICK H. W., SLAUGHTER D. P., HINKAMP J. F. (1963). MALIGNANT MELANOMAS OF THE SKIN OF THE HEAD AND NECK.. Am J Surg.

[OCR_00760] Shaw H. M., Balch C. M., Soong S. J., Milton G. W., McCarthy W. H. (1985). Prognostic histopathological factors in malignant melanoma.. Pathology.

[OCR_00765] Simons J. N. (1972). Malignant melanoma of the head and neck.. Am J Surg.

[OCR_00769] Sober A. J., Fitzpatrick T. B., Mihm M. C. (1980). Primary melanoma of the skin: recognition and management.. J Am Acad Dermatol.

[OCR_00785] Urist M. M., Balch C. M., Soong S. J., Milton G. W., Shaw H. M., McGovern V. J., Murad T. M., McCarthy W. H., Maddox W. A. (1984). Head and neck melanoma in 534 clinical Stage I patients. A prognostic factors analysis and results of surgical treatment.. Ann Surg.

[OCR_00790] Van Der Esch E. P., Cascinelli N., Preda F., Morabito A., Bufalino R. (1981). Stage I melanoma of the skin: evaluation of prognosis according to histologic characteristics.. Cancer.

[OCR_00796] Wanebo H. J., Cooper P. H., Young D. V., Harpole D. H., Kaiser D. L. (1988). Prognostic factors in head and neck melanoma. Effect of lesion location.. Cancer.

